# Astrocytic Regulation of Synchronous Bursting in Cortical Cultures: From Local to Global

**DOI:** 10.1093/texcom/tgaa053

**Published:** 2020-08-24

**Authors:** Ravi Kumar, Yu-Ting Huang, Chun-Chung Chen, Shun-Fen Tzeng, Chi-Keung Chan

**Affiliations:** Taiwan International Graduate Program in Interdisciplinary Neuroscience, National Cheng Kung University and Academia Sinica, Taipei 115, Taiwan, R.O.C; Institute of Physics, Academia Sinica, Taipei 115, Taiwan, R.O.C; Institute of Physics, Academia Sinica, Taipei 115, Taiwan, R.O.C; Institute of Physics, Academia Sinica, Taipei 115, Taiwan, R.O.C; Department of Life Sciences, National Cheng Kung University, Tainan 70101, Taiwan, R.O.C; Institute of Physics, Academia Sinica, Taipei 115, Taiwan, R.O.C

**Keywords:** glutamate trafficking, network dynamics, neuron–glia interaction, simulation, tripartite synapse

## Abstract

Synchronous bursting (SB) is ubiquitous in neuronal networks and independent of network structure. Although it is known to be driven by glutamatergic neurotransmissions, its underlying mechanism remains unclear. Recent studies show that local glutamate recycle by astrocytes affects nearby neuronal activities, which indicate that the local dynamics might also be the origin of SBs in networks. We investigated the effects of local glutamate dynamics on SBs in both cultures developed on multielectrode array (MEA) systems and a tripartite synapse simulation. Local glutamate uptake by astrocytes was altered by pharmacological targeting of GLT-1 glutamate transporters, whereas neuronal firing activities and synaptic glutamate level was simultaneously monitored with MEA and astrocyte-specific glutamate sensors (intensity-based glutamate-sensing fluorescent reporter), respectively. Global SB properties were significantly altered on targeting GLT-1. Detailed simulation of a network with astrocytic glutamate uptake and recycle mechanisms, conforming with the experimental observations, shows that astrocytes function as a slow negative feedback to neuronal activities in the network. SB in the network can be realized as an alternation between positive and negative feedback in the neurons and astrocytes, respectively. An understanding of glutamate trafficking dynamics is of general application to explain how astrocyte malfunction can result in pathological seizure-like phenomena in neuronal systems.

## Introduction

Synchronous firings of neurons are frequent in brains that can emerge as patterns of low-frequency spiking or synchronous bursting (SB), where the SBs are believed to produce functions of a network (Arenas et al. 2008). Although astrocytes and neurons both constitute our brains, the SBs are commonly considered to result from neuron–neuron interactions alone (von Krosigk et al. 1993; Sirota et al. 2008; Penn et al. 2016). However, astrocytes are known to modulate nearby neuronal activities by forming tripartite synapses and exhibiting calcium excitability in response to surrounding neuronal activities (Araque et al. 1999; Pascual et al. 2005). Therefore, a possible astrocytic mechanism for SB cannot be ruled out. Recent findings show that astrocytes play an important role in shaping neuronal firings by regulating synaptic glutamate via GLT-1 glutamate transporters (GluTs) (Murphy-Royal et al. 2015), providing evidence to a possible astrocytic mechanism in SB. However, it still remains unclear how the local properties of a synapse relate to the SB phenomenon, which is a network behavior.

Since SBs are ubiquitous in both structured brains (Hutchison et al. 2004; Axmacher et al. 2006) as well as randomly connected cortical cultures (Wagenaar et al. 2006; Adams et al. 2011), local astrocytic modulation of neuronal firings possibly could be the basic mechanism for SB generation in a network, independent of connection topologies. In the SB phenomena, the neuronal system can be described to alternate between 2 states: “active state” (during an SB) and “dormant state” (time between SBs). Intuitively, the pools of ready-to-be-released glutamate in the neurons must deplete during the “active” state and undergo recovery in the “dormant” state. Our view of SB mechanism is that this alternation between pool depletion and replenishment is intimately related to the recycling of glutamate in the tripartite synapses formed by neurons and astrocytes in the network.

In a traditional bipartite synapse model, as proposed by Tsodyks, Uziel, and Markram (TUM), neuronal excitability is characterized by recycling of a conserved amount of synaptic resources (Tsodyks et al. 2000). In this TUM model, synaptic resources (i.e., glutamate) are recycled through 3 states: recovered, active, and inactive. It is the recovered state that provides excitability to neurons. However, experiments have shown that released glutamate are uptaken by both neurons and astrocytes, and 80–90% of the released glutamate are uptaken by astrocytes (Danbolt et al. 1998), which were not considered in the TUM model. Recently, astrocytic GLT-1 was found to regulate synaptic currents (Murphy-Royal et al. 2015) and the period of SBs (Huang et al. 2017a). It is also known that glutamate transported into the astrocytes get converted into glutamine and transported to neurons as precursors for glutamate, namely glutamate–glutamine cycle (Danbolt et al. 1998; Hertz et al. 1999). Since the timescale of astrocytic glutamate recycling pathway is much longer than the presynaptic neuronal pathway, the recycle timescale difference should generate multiple timescales of glutamate depression. To account for the multiple timescales in SB, Volman et al. (2007) extended the TUM model by introducing a hypothetical “superinactive” state for glutamate. In our view, this superinactive state could just be the glutamine in the astrocyte, consistent with the findings of Tani et al. (2014); Bacci et al. (2002), which show direct evidence on the necessity of synaptically localized glutamate–glutamine cycle for glutamate synthesis at excitatory terminals. These lines of reasoning strongly support that SB could be the consequence of astrocyte-mediated glutamate regulation in response to neuronal dynamics.

We hypothesized that SBs in neuronal networks arise from the local effects of the glutamate recycling in the tripartite synapses. We tested this hypothesis by both experiments and numerical simulation. In experiments, GluTs were targeted in cortical cultures developed on multielectrode array (MEA). Combining astrocyte-specific fluorescent probes with MEA, responses from neurons and astrocytes were recorded simultaneously. To test the validity of astrocytic regulation of SB, we developed a tripartite synapse model to include astrocytes in the glutamate recycling process. Results from both our experiments and simulations were found consistent with our hypothesis. Our novel finding suggests that the forms of SB are governed by the amount of glutamate residing in astrocytes. This later fact can be used to understand the epileptic form of firings (SB) in GLT-1 dysfunction (Rothstein et al. 1996; Coulter and Eid 2012).

## Materials and Methods

All samples from animals were prepared according to the guidelines approved by the Academia Sinica IACUC (Protocol: 12-12-475). All pharmacological experiments and simultaneous recordings of MEA and intensity-based glutamate-sensing fluorescent reporter (iGluSnFr) glutamate imaging/GCaMP6f-calcium imaging were performed on cultures (age *>* 20 days in vitro [DIV]) inside home-made incubation chamber maintained at 5% CO_2_ and 95% air at 37°C.

### Cell Culture

Cell culture protocol was adapted from Potter and DeMarse (2001). Briefly, neocortical tissues were obtained from postnatal mice pups (P0) FVB/NJ-wild type under sterile conditions. Pieces of dissected cortices were first enzymatically dissociated at 37°C with 0.125% trypsin solution for 10 min and then mechanically triturated with sterile and fire-polished Pasteur glass pipette. From this cell suspension, cells were plated on 4-well MEA probes at 2000 cells/mm^2^. The surfaces of the plating areas were previously coated with 0.1% polyethylenimine and laminin for at least 2 h. Cells in the remaining suspension were seeded into flasks and cultured simultaneously for obtaining conditioned media (CM). All the cultured cells were maintained in NeuroBasal-A medium (Invitrogen) supplemented with GlutaMax and B27, inside a humidified incubator with 5% CO_2_ and 95% air at 37°C. To prevent evaporation and reduce chances of contamination, culture wells were always sealed with home-made Teflon membrane caps. From each well (total volume capacity, 250 μL), half (125 μL) of the maintenance media was replaced with fresh maintenance media in every 3 days.

### Adeno-Associated Virus Infection

At the age of 7 DIV, selected developing cortical networks were infected with AAV.GFAP.iGluSnFR to express glial fibrillary acidic protein (GFAP)-promoted glutamate sensors on astrocytes. The virus pENN.AAV.GFAP.iGluSnFr.WPRE.SV40 was a gift from Loren Looger (Addgene plasmid cat. #98930). At the same time, few other growing cortical networks were virally infected with gfaABC1D-driven (similar to GFAP promoter) cyto-GCaMP6f to express calcium sensors in astrocytes. The virus pZac2.1 gfaABC1D-cyto-GCaMP6f was a gift from Baljit Khakh (Addgene plasmid cat. #52925). Viral infections were performed at up to 2 × 10^10^ viral particles as final concentration in 200 μL volume per well. In total, 24 h after infection, samples were washed with CM and maintained in the usual manner for more than 2 weeks before performing any fluorescence imaging or pharmacological experiment.

### Pharmacology

All the drugs used in this study were purchased from Tocris Bioscience unless otherwise noted. To obtain more reproducible results from drug experiments, the following protocol was adapted from Keefer et al. (2001). Briefly, prior to any pharmacology experiment the maintenance media of test sample well was completely replaced and bathed with 200 μL CM (pooled from flask cultures). Following a 30 min of stabilization, a baseline activities of the test sample was recorded for the next 30 min. Test drug was then added (maximum volume up to 2 μL), gently mixed by pipetting through micropipette. After drug addition, cultures were again left to stabilize for the next 10 min. Influence of test drugs on network firing activities were usually visible within 5 min of addition. Steady state response of the network was then recorded. Drugs were washed with the same CM 3 times within a span of 5 min. After another 30 min of stabilization, recovered activities were recorded. The drugs and their final concentrations used: dihydrokainic acid (DHK, GLT-1 inhibitor, 200 μM, cat. no. #0111); DL-threo-beta-benzyloxyaspartate (DL-TBOA, nonselective excitatory amino acid transporter [EAAT] inhibitor, 10 μM, cat. no. #1223); GT949 (positive allosteric modulator [PAM] of EAAT2, 10 μM, cat. no. #6578); bicuculline methiodide (Bic, γ-aminobutyric acid [GABA] antagonist, 10 μM, Abcam, cat. no. #ab120108; riluzole hydrochloride [Na^+^ channel inhibitor], 10 μM, cat. no. #0768).

### Electrophysiological Recording

Cultures were developed on 4-well multielectrode planar arrays purchased from Qwane Biosciences (MEA-60-4well-PtB). Each of the 4 wells were embedded with 14 platinum black electrodes laid out in a square grid. The electrodes (diameter 30 μm each) were separated by 200 μm. Electrical signals from active cortical networks were sampled at 20 kHz, amplified using MEA-60 preamplifier and digitized using MC Card PCI board in vitro MEA system (MEA1060-Inv-BC, Multi Channel Systems MCS GmbH). Electrode configuration settings and data acquisition were performed with MEA Select and MC Rack software (both Multi Channel Systems MCS GmbH), respectively. For long duration recording (up to several hours), an incubation chamber was constructed around the MEA system consisting of a temperature controller maintaining 37°C and CO_2_ supply. The recording media was the same as the CM as it allows long-term recording for several hours without compromising with the health of cells in the network. As CM contained GlutaMax, its effects on SB were tested by comparing the activity patterns of the same network in balanced salt solution (with 0.8 mM Mg^+2^). It was observed that in presence of GlutaMax, cultures showed noisier SB patterns that could be reduced while SB detection during data preprocessing as described below. All pharmacological experiments were performed on mature samples (*>*20 DIV) that showed stable array-wide SB events.

### Fluorescence Microscopy

iGluSnFr or GCaMP6f imaging was performed inside a custom home-made incubation chamber built on a phase-contrast microscope platform for maintaining 5% CO_2_ and 95% air at 37°C. For obtaining higher sensitivity and good signal to noise ratio for longer continuous recording (up to 15 min per session), a CCD camera (Prosilica GE680, Allied Vision) was coupled with an image intensifier (II18, Lambert Instruments). For epifluorescence illumination, a mercury arc lamp illuminator (X-cite, Lumen Dynamics) was used. Calcium/glutamate fluorescence signals from test samples were captured with 640 × 480 resolution at 25–100 Hz sampling rate on ×20 objective, covering an area of ≈0.3 mm^2^.

### Immunocytochemistry

After performing experiments, selected samples were fixed with 4% paraformaldehyde solution for immunocytochemistry. Neuronal soma and dendrites were identified with microtubule-associated protein 2 (MAP2, Sigma, cat no. #M4403). Neuronal axon projections and their connectivity was identified with neuron-specific class III β-tubulin (Tuj1, GeneTex, cat. no. #GTX85469) primary antibody. For identifying astrocytes and GluT (GLT-1) expression, GFAP (Sigma, cat. no. #G3893) and GLT-1 marker antibody (Invitrogen, cat. no. #701 988) were used, respectively. Nuclei of all cell types were identified with 4′,6-diamidino-2-phenylindole (DAPI) staining. Confocal imaging of immunostained samples was performed at ×10/×20 magnification using inverted confocal microscope (ZEISS LSM 880 system).

### Analysis

Data analysis was performed using MATLAB (MathWorks), ImageJ (National Institutes of Health—public domain), and Prism 8 (GraphPad) software.

#### MEA Data Preprocessing

Data acquired from Multi Channel Systems recordings were imported into MATLAB for further processing and analysis. Before spike detection, raw data were preprocessed through a Butterworth second-order high-pass filter, with cutoff frequency (200 Hz) to remove low-frequency line and other system-generated noise. Data presented here are not spike sorted. For each well, spike times from all the 14 channels were detected using Precise Timing Spike Detection algorithm (Maccione et al. 2009) and arranged in ascending order (*t*_1_*, t*_2_*,..., t_N_*).

#### SB Detection

SBs were detected using the method described in (Huang et al. 2017b). Briefly, firing rate time histogram of all channels combined was generated with a bin size of 10 ms. For every network its maximum firing rate (*R*_max_) was determined. Two threshold criteria (a lower ε*R*_max_ followed by an upper ∆*R*_max_) were required to be satisfied, where ε = 0.04 and ∆ = 0.2 typically. The lower threshold decided whether the active state in the network is initiated. Starting of a burst is registered at *t_s_* once the network becomes active at *t_s_* and remains active until its summed spike rate reaches its upper threshold. Burst end (*t_e_*) is registered when the network becomes inactive and remains inactive at least for a duration of 1 s (τ_rest_). After post hoc manual inspection, ε*,* ∆, and τ_rest_ were slightly tuned whenever it was required to increase the efficiency of SB detection. To compare the level of SB activities across different networks “SB index” was defined, as the ratio of sum of spikes detected within all SBs to the sum of all spikes detected array-wide in a recording, that is, $\frac{\sum \limits_{i=1}^M\sum \limits_{j={t}_s^i}^{j={t}_e^i}{t}_j^i}{\sum \limits_{j=1}^N{t}_j}$ where *t_j_* is a detected spike time, *N* is the total number of spikes detected, and *M* is total number of SBs detected. An SB index of 0.5 indicates that 50% of array-wide spiking activities occurred in the form of SBs.

#### Fluorescence Imaging Data Preprocessing and Analysis

An instantaneous fluorescence intensity (*F_t_*)—time series was generated for each recording using “Plot *Z*-axis Profile,” ImageJ plugin. For each frame, *F_t_* was calculated by averaging its pixel values. Further analyses were carried out in MATLAB. Drifts and photo-bleaching trend lines were removed using a polynomial curve fitting method, where a fitted curve was subtracted to remove any trend. Change in fluorescence ∆*F/F* was then defined as (*F_t_* − *F*)*/F*, where *F* represented the mean instantaneous fluorescence intensity of the recording.

### Tripartite Synapse TUMA Model

Our tripartite TUMA model extends on the short-term synaptic depression model described by TUM (Tsodyks and Markram 1997; Tsodyks et al. 2000), hence referred to as TUM model, to consider the effects of astrocyte. The TUM model implements 3 states of synaptic resource trafficking with neuronal transmitters represented by 3 variables: *X*, *Y*, and *Z*, corresponding to the fractions of the resource in the recovered, active, and inactive states, respectively. Assuming synaptic resource to be a conserved quantity, the relation *X* + *Y* + *Z* = 1 was held. From here on, we will refer to synaptic resource as glutamate. Upon spike arrival at time *t* = *t*_spk_, the transitions of glutamate between the 3 states are given by the following 3 equations,

(1) \begin{equation*} \frac{dX}{dt}=\frac{Z}{\tau_r}- uX\delta \left(t-{t}_{spk}\right) \end{equation*}

(2) \begin{equation*} \frac{dY}{dt}= uX\delta \left(t-{t}_{spk}\right)-\frac{Y}{\tau_d} \end{equation*}

(3) \begin{equation*} \frac{dZ}{dt}=\frac{Y}{\tau_d}-\frac{Z}{\tau_r} \end{equation*}

Equations 1–3 describe the transportation, transformation, and conservation of the synaptic glutamate under the spiking of the presynaptic neuron, with *u* being the facilitation factor provided from calcium influx. The relevant timescales are τ*_r_*, as the recovery time for the transformation from *Z* to *X*, and τ*_d_*, as the decay time from *Y* to *Z* are phenomenological. This model is shown to well reproduce the synaptic responses between pyramidal neurons (Tsodyks and Markram 1997). When an action potential arrives at the TUM synapse, the presynaptic cell will release a fraction *uX* of the neural transmitters into the synapse and this amount of transmitters will bind to the receptors to become *Y* and produce an excitatory postsynaptic current (EPSC) in the postsynaptic cell (Clements et al. 1992; Bredt and Nicoll 2003). Physiologically, τ*_d_* represents the uptake process of the active transmitters back into the presynaptic cell to become *Z*, whereas τ*_r_* represents the repackaging process of the neural transmitters so that they can be in the ready-to-release pool, *X*, again (Rizzoli and Betz 2005; Alabi and Tsien 2012).

In the TUM picture, the clearance of the glutamate from the synapse is carried out through the uptake by the presynaptic cell. However, it is known that fast glutamate uptakes by GluTs are carried out both at presynapse cell and mostly through astrocytic processes to ensure a rapid clearance and thus decay of the EPSCs (Danbolt 2001). In order to include the effects of astrocytes on the clearance process, we introduced a new pathway as well as a new state to the TUM model allowing the uptake of the synaptic glutamate by the astrocytes. This is referred to as the TUMA model as described in the Figure 6*A*. The state *A* is the fraction of glutamate in the astrocytes. From the diagram, glutamate in *A* comes from *Y* in the synapse and can be transformed into *Z* and then finally to *X*. The timescales τ_au_, τ_nu_, and τ*_g_* are for the astrocytic uptake, the neuronal uptake, and the inactivation in the *A* state, respectively. In this model, the released glutamate (*Y*) is transformed back into *Z* through 2 modes: a fast and a slow recycle pathways. The fast path takes place through direct presynaptic neuronal uptake of glutamate from the cleft within a timescale of τ_nu_ by EAAT3 (EAAC1) group of GluTs (Danbolt 2001). For the slow path, glutamate in the synapse are first uptaken by the astrocytes via EAAT2 (GLT-1) group of GluTs with a timescale of τ_au_. These uptaken glutamate in astrocytes are then converted into neutral molecules, glutamine (Waniewski and Martin 1986; Broer and Brookes 2001) and then converted back to the glutamate (Bak et al. 2006) once they are transported back into the presynaptic cell (*Z*). This whole process of glutamate conversion, release, uptake, and reconversion to glutamate (alternatively, glutamate–glutamine cycle) is slow and represented by a long timescale of inactivation τ*_g_*. Therefore, one can expect ${\tau}_g\gg \tau \mathrm{nu}$ and τ_au_. The “recovered” glutamate at the presynapses in *Z* state are now readily loaded into vesicles and retrieved into active zone within τ*_r_*. This process corresponds with the vesicle retrieval and recycle phenomenon. In the “kiss-and-run” mode of vesicle retrieval, vesicle recycle in presynaptic cell occurs at timescale within the range of 400–860 ms (Gandhi and Stevens, 2003). In our model, the recycling timescale parameter τ*_r_* is created to represent all the slow processes from *Z* (recovered) state of glutamate to the *X* (ready to release) state. Therefore, the τ*_r_* here is being used as a lumped parameter. The mechanism described thus takes the following form of equations:

(4) \begin{equation*} \frac{dX}{dt}=\frac{Z}{\tau_r}- uX\delta \left(t-{t}_{spk}\right)-\xi X \end{equation*}

(5) \begin{equation*} \frac{dY}{dt}= uX\delta \left(t-{t}_{spk}\right)-\frac{Y}{\tau_{nu}}-\frac{Y}{\tau_{au}}+\xi X \end{equation*}

(6) \begin{equation*} \frac{dZ}{dt}=\frac{Y}{\tau_{nu}}+\frac{A}{\tau_g}-\frac{Z}{\tau_r} \end{equation*}

(7) \begin{equation*} \frac{dA}{dt}=\frac{Y}{\tau_{au}}-\frac{A}{\tau_g} \end{equation*}

Note that the term ξ*X* is added to the Equation 4 because it is known that glutamate can be released either spontaneously or triggered by an action potential. The noise term for the asynchronous release as an extension to the TUM model was first studied by Volman et al. (2007). The goal of Volman’s work was to understand the dynamics of reverberation in neuronal cultures through reproduction of the experimental observations in the model with calcium dynamics. The noise term added to equation 4 was identified as spontaneous release events driven by the presynaptic residual calcium dynamics. Similar to Volman et al. (2007), the calcium-dependent asynchronous release was found to be essential for the model to reproduce the experimental observations. Therefore, we adopted their mechanism for the construction of TUMA model.

### Simulation

#### Neuronal Model

Similar to Volman et al. (2007), we implemented Morris–Lecar (ML) neurons (Morris and Lecar 1981) to test our synaptic model. The dynamics of ML neurons are described by,

(8) \begin{equation*} C\frac{dV}{dt}=-{I}_{\mathrm{ion}}+{I}_{\mathrm{syn}}+{I}_{\mathrm{bg}} \end{equation*}

where *V* denotes membrane potential, *C* is membrane capacity, *I*_ion_ is sum of *V*-, *t*-dependent currents through the various ionic channel types, *I*_syn_ is the current generated from synaptic activities and *I*_bg_ is the external input current. The *I*_ion_ here is the sum of current through membrane from the Ca^2+^, K^+^ and leakage currents with their corresponding channel conductivity denoted *g*_Ca_, *g_K_*, and *g_L_* taken as constants.

(9) \begin{equation*} {I}_{\mathrm{ion}}={g}_{\mathrm{Ca}}{m}_{\infty}\left(V-{V}_{\mathrm{ca}}\right)+{g}_kW\left(V-{V}_k\right)+{g}_L\left(V-{V}_L\right) \end{equation*}

*W* describes the voltage-dependent fraction of potassium channels in various conducting states (e.g., open or closed) at time *t*.

(10) \begin{equation*} \frac{dW}{dt}=\theta \frac{W_{\infty }(V)-W(V)}{\tau_W(V)} \end{equation*}

(11) \begin{equation*} {\tau}_W={\left(\mathit{\cosh}\left(\frac{V-{V}_3}{2{V}_4}\right)\right)}^{-1} \end{equation*}

Here, the time constant related to potassium channel is described as τ*_W_* and υ is a temperature-like parameter. The steady-state fraction of open potassium and calcium channels are described by

(12) \begin{equation*} {W}_{\infty }=\frac{1}{2}\left(1+\tan h\left(\frac{V-{V}_3}{V_4}\right)\right) \end{equation*}

(13) \begin{equation*} {m}_{\infty }=\frac{1}{2}\left(1+\tan h\left(\frac{V-{V}_1}{V_2}\right)\right) \end{equation*}

The postsynaptic current generated at synapse, as described in equations (4–7), is represented by the *I*_syn_ term.

(14) \begin{equation*} {I}_{\mathrm{syn}}=G\left({V}_r-V\right) \end{equation*}

where membrane conductance of the postsynaptic neuron is denoted by *G* and its reversal potential by *V_r_*. Another threshold parameter *V*_th_ of membrane potential was added to define the spiking events resulting in synchronous releases of neural transmitters at efferent synapses. Additionally, a residual cytosolic calcium variable *R*_Ca_ driven by the spiking events, as described by Volman et al. (2007), was also incorporated to produce reverberatory dynamics observed in cortical networks. The dynamics of residual calcium *R*_Ca_ is described by the equation,

(15) \begin{equation*} \frac{dR_{\mathrm{Ca}}}{dt}=\frac{-\beta{R}_{\mathrm{Ca}}^n}{\kappa_r^n+{R}_{\mathrm{Ca}}^n}+{I}_p+\mathrm{S}\gamma \mathrm{log}\frac{R_{\mathrm{Ca}}^0}{R_{\mathrm{Ca}}} \end{equation*}

where the first term describes action of calcium pumps expelling calcium from cytoplasm to extracellular space, *I_p_* is the passive flux of calcium into cytosol, and the third term corresponds to the influx of calcium induced by the spike train $S={\sum}_{\sigma}\delta \Big(t-{t}_{\sigma}\Big)$ in which *t*_σ_ is the spike time. The residual calcium is used to determine the rate,

(16) \begin{equation*} \eta{R}_{\mathrm{Ca}}={\eta}_{\mathrm{max}}\frac{R_{\mathrm{Ca}}^m}{\kappa_a^m+{R}_{\mathrm{Ca}}^m} \end{equation*}

of synapse-dependent neural transmitter asynchronous releases following an independent Poisson process at each efferent synapse (Huang et al. 2017b). Here, η(*R*_Ca_) is the probability of asynchronous release and η_max_ is the maximal rate of asynchronous release. Once the neural transmitters release is induced by the spike-driven synchronous and calcium-dependent asynchronous events, a 4-state decaying dynamics is followed based on the TUMA synapse description (eqs 4–7). In equation (4), the variable $\xi =\overline{\xi}{\sum}_a\delta \Big(t-{t}_a\Big)$ is then a summation of the asynchronous release events *a* with the Poisson rate given by equation 15. The values of parameters used are listed in the Table 3.

#### Network Topology and Connectivity

To investigate the glutamate dynamics around synapses, a network of 100 ML randomly connected (connection probability = 0.1) with each other through the TUMA synapses was constructed. The inhibitory-to-excitatory ratio for the neurons was set to 0.2 (Soriano et al. 2008). The postsynaptic membrane conductance (*G*) is driven by the fractions of neurotransmitters in the active state *Y* and synaptic weight (*w* = 4), a scale for the density of postsynaptic effectors such as glutamate receptors. For the *i*-th neuron, the conductance is the summation of all the contribution of *Y_j,i_* with weight *w_j,i_* from neighboring neurons *j*:

(17) \begin{equation*} {G}_i=\sum_j{w}_{i,j}{Y}_{j,i}\left(1-b{Y}_{j,i}\right) \end{equation*}

Here, we assume the postsynaptic effects are given by the factor (1 − *bY_j,i_*). The parameter *b* introduced here represents current saturation or receptor desensitization effect at the postsynaptic cell. The fraction of glutamate in *Y* transient during SBs is always very small (*<*0.1), which is due to the fast rate of clearance of the synapse by the uptake processes. In simulation, we found that the nonlinear effects are significant only when *b* is quite large. When 0 ≤ *b <* 1, *bY_j,i_* becomes an even smaller quantity making the postsynaptic nonlinear effect almost negligible. Therefore, the effect of postsynaptic saturation was ignored and the results reported below were obtained with *b* = 0. The synaptic weights (*w*) were randomly drawn from a Gaussian distribution truncated at its width that is set to ±20% of its mean (*w* = 4) for the connected neurons.

#### Implementation and Code Accessibility

A GUI program, written in C++ programming language using the Common Simulation Tools framework in previous work of (Huang et al. 2017b) implementing the Volman’s model, was used to perform the simulations. The program was modified as per our TUMA synaptic dynamics for the simulations. Original package measim implementing Volman’s synaptic model is available on GitHub (https://github.com/chnchg/measim). Spike data obtained from the simulation were exported to the MATLAB software for further processing and analysis. The same algorithm for SB detection (described above) was used for comparison with experimental results.

### Statistical Analysis

Assumptions of normality were validated using D’Agostino–Pearson omnibus test (α = 0*.*05). Hypothesis testing was performed on repeated measures using parametric 1-way ANOVA (analysis of variance) or 2-tailed paired/unpaired *t* tests. In case of 1-way ANOVA in multiple groups, Tukey’s post hoc multiple-comparison tests were also performed. Nonparametric Friedman test was performed for multiple group comparison in case any one group failed the normality test. Sample sizes are listed in the figure captions. Error bars indicate standard error of the mean unless otherwise specified.

## Results

### SB-Associated Active and Dormant States in Cortical Networks

**Figure 1:**
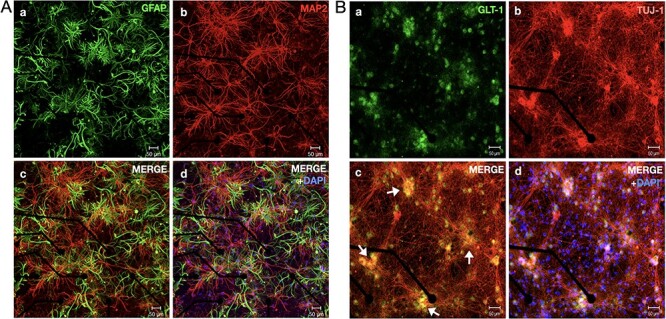
Astrocytes and neurons develop into complex networks in cortical cultures. Confocal micrograph of a 22-DIV-old cortical network fixed on MEAs is shown here. (*A*-*a*) GFAP immunostained (green) cortical network shows highly complex dense structures of stellate type astrocytes coexisting with highly interconnected network of MAP2-stained (red) neurons (*A*-*b*). (*A*-*c*) Merged image of GFAP and MAP2 staining shows tightly interlinked neuron-astrocyte *network. (A*-*d*) shows colocalized image along with DAPI signals. (*B*) Confocal micrograph of a 14-DIV-old fixed cortical network fixed on a sample MEA. (*B*-*a*) shows immunostained signals of GLT-1 (green) GluTs distributed as clusters. (*B*-*b*) Neurons were stained for Tuj-1 (red), a marker for neuronal cytoskeleton. Neurons can be seen to be randomly organized as thick clusters with hub-like topology. (*B*-*c*) Merged GLT-1 and Tuj-1 staining signals show that GLT-1 was strongly expressed and localized at the hub locations where neuronal cell bodies form dense synaptic clusters (arrows). (*B*-*d*) shows colocalized image along with DAPI signals.

**Figure 2:**
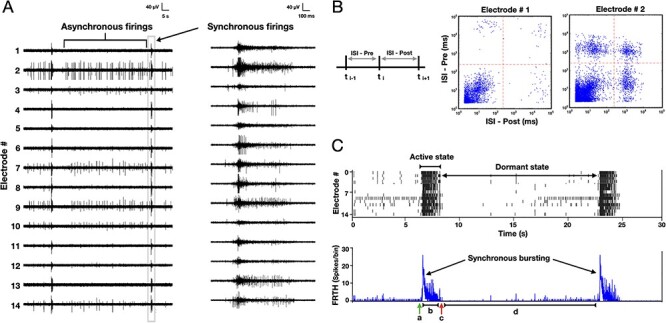
Active and dormant states of neurons in cortical culture. Time series of extracellular field potentials of a sample cortical network recorded with 14 electrodes using an MEA probe is shown here. Spontaneous electrical activities of cortical neurons in a developing network (22 DIV) appear as asynchronous or synchronous firings. (*B*) An ISI distribution, generated by plotting time intervals (top) between successive firings, shows a distinction between synchronous and asynchronous firing events. Electrode #1 engaging only in synchronous firings exhibit only 1 dense cluster of ISI lying in 2–100-ms range (middle), whereas the electrode #2 also engaging in asynchronous firings shows additional ISI clusters lying above 1-s region (bottom). (*C*) Raster plot of detected spikes (top) illustrates 2 states of network in general: “active” state when all units exhibit SB firings followed by a “dormant” state in which most units show no to very few activities. Corresponding FRTH (bottom trace), calculated by summing all the detected spikes within 5-ms bin size, displays the kinetics of the states in the network. The kinetics involve an initiation (*a*), maintenance (*b*), and termination (*c*) phases followed by a much longer dormant phase (*d*).

Neurons in cortical cell cultures develop into self-organized networks forming recurrent connections (Fig. 1*A*). At the same time, astrocytes also develop by extending their processes to form their own complex network and intimately connected with the neuronal network. Functionally, the neurons exhibit a wide range of spontaneous firing patterns (Wagenaar et al. 2006) during their course of development. Typically, after 3 weeks of age neuronal connections become functionally mature, and the array-wide firing patterns turn stable. Each electrode of MEA generally display 2 patterns of neuronal activities: either bursts of spikes followed by isolated spikes (ch#2,3,7,9,10,14 in Fig. 2*A*), or only bursts of spikes (ch#1,4,5,6,8,11,12,13). Aligning the temporal activities in all the electrodes, the overall array-wide firing dynamics thus appears as either asynchronous or synchronous firings in a network. A distinction between SB and asynchronous spiking events can be made by generating interspike interval (ISI, time interval between successive discharge) return map (Fig. 2*B*). Electrodes that exhibit only synchronous events, their ISI return map show single cluster lying within the range of 2–250 ms. Whereas electrodes that also show asynchronous firings, display additional clusters of ISI distributed in the interval between 1 and 10 s. We will refer to the synchronous discharge state of network as the “active state,” since all neurons collectively remain active during this state (Fig. 2*C*). Within an active state a network exhibits different kinetics, as shown in the firing-rate time-histogram (FRTH) plot. FRTH was generated by summing the total number of array-wide action potentials detected in a 5-ms bin size. A generic kinetic structure of SB consists of initiation, maintenance, termination, and dormant phases (Fig. 2*C*, bottom). Initiation of SB occurs by assembly of neuronal activities at an exponential rate (Eytan and Marom 2006). Postassembly of neuronal firings, array-wide firing reaches a peak level then gradually decays to the extent where all neuronal spikes ceases completely. By taking reciprocal of the ISI ranges stated above, it can be seen that in unsorted spike data from single electrode, the firing rate during the active state can range between 10 and 500 Hz. After termination of the SB, the network maintains a period of quiescence for several seconds and regains its basal firing activities until next SB occurs. We will refer to the time between the termination and the initiation of the next SB as the “dormant state” of the network. During the dormant state some neurons may still exhibit spontaneous firings at a rate within the range of 0.1–4 Hz.

### Synaptic Glutamate Dynamics Accompanying SB

**Figure 3:**
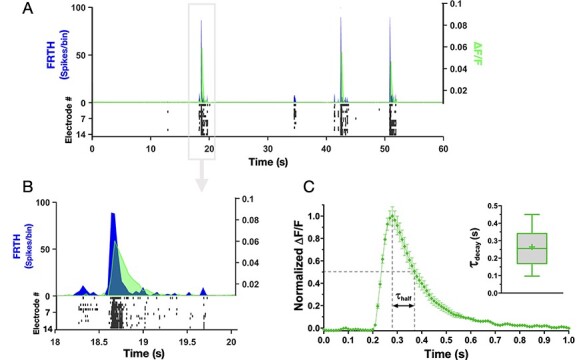
Synaptic glutamate level rises and falls during SBs. Simultaneous recording of neuronal activities with MEA and synaptic glutamate dynamics detected by astrocytes with iGluSnFr imaging is shown here. (*A*) Top panel shows summed neuronal spiking activities as FRTH (with bin size 40 ms, black trace) overlapped with glutamate dynamics detected simultaneously through iGluSnFr imaging. Change in fluorescence is plotted as ∆*F/F* (gray trace). Bottom raster plot (black) shows detected neuronal activities from individual electrodes. (*B*) shows enlarged view of one of the SB events in A. Note that synaptic glutamate level increases globally and decays rapidly during and after SB events. (*C*) Shows average normalized fluorescence ∆*F/F* from a sample network. The time taken for decrease of synaptic glutamate level from its maximum to half during SB is denoted by τ_half_. Inset shows summary of glutamate decay time (τ_decay_) in cultured cortical networks, calculated as τ_half_*/ln2*. Middle line in the box plot shows median and cross represents mean (0.26 ± 0.11 s, *n* = 8 cultures).

Firing dynamics observed in SB involves fast recurrent excitation of α-amino-3-hydroxy-5-methyl-4-isoxazolepropionic acid (AMPA) and N-methyl-D-aspartate (NMDA) receptors by synapse-released glutamate (Clements et al. 1992; Bredt and Nicoll 2003). Previous studies indicate that most of the synaptic glutamate (≈80%) is uptaken by astrocytic processes through GLT-1 transporters while only a smaller portion is directly uptaken by the neurons (Lehre and Danbolt 1998; Eulenburg and Gomeza 2010). This distinction is due to the difference in the number of transporter expressions on the membranes of the 2 cell types. We performed a qualitative check on GLT-1 expression in our sample cortical cultures. Confocal imaging revealed a highly clustered expression of GLT-1 (Fig. 1*B*) around hub-like clustered neuronal bodies. It is known that these neuronal hub structures are composed of organized synaptic clusters formed by assembly of presynaptic, postsynaptic, and dendritic structures (Kavalali et al. 1999). Observation of intense GLT-1 expressions implies high glutamate activities at these locations. Our goal was to detect glutamate dynamics during SBs in the immediate surrounding of the astrocytes. Therefore, we employed glutamate sensors genetically expressed on astrocytic membrane facing extracellular space. Astrocytes in developing cortical cultures were infected with adeno-associated viruses expressing GFAP-targeted iGluSnFr (Marvin et al. 2013) (see Material and Methods for details). This approach has been previously shown to indicate the kinetics of glutamate clearance by astrocytes (Armbruster et al. 2016). Therefore, we implemented this method for evaluating GluT activities during SBs. Within 2 weeks (postinfection), fluorescence signals from astrocytes were detectable. Simultaneous recording of MEA and whole-field synaptic glutamate imaging revealed sharp elevation of iGluSnFr signals in association with SB events recorded with MEA (Fig. 3*A*). Its kinetics almost overlapped with the time course of the active state in neuronal network. Evidently, the sharp rise in glutamate signal was due to synaptic releases of glutamate during intense burst firings by neurons. Notably, the glutamate level rapidly decayed back to baseline level immediately toward the termination of the SBs (Fig. 3*B*). Detected iGluSnFr signal continued to remain at its baseline level until the next SB occurred. The time taken for the decrease of glutamate from its maximum level to half during SB was defined as τ_half_. The glutamate decay time τ_decay_ (assuming exponential decay function; Armbruster et al. 2016) was computed as τ_half_*/*ln2 (Fig. 3*C*) to reflect the glutamate clearance time by the astrocytes. In our culture system, the decay time was found to be distributed around 0.26 ± 0.11 s (*N* = 8 cultures).

### Pharmacologically Targeting Astrocytic GLT-1 Alters SB Properties

**Figure 4:**
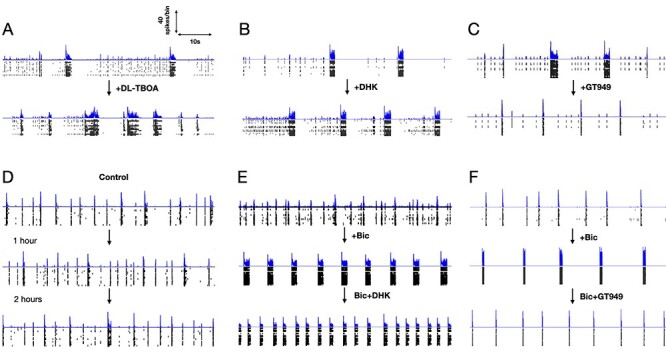
Pharmacological manipulation of GluTs alters SB dynamics. (*A*–*F*) shows 1-min snapshots of firing activities obtained from 6 different cortical networks. FRTHs generated with a bin size of 5 ms are shown above the raster plot of detected spikes. The intervals between each snapshot of a network, shown in the order as indicated by the arrow marks, were at least separated by 45 min. The first snapshot of each network shows its reference activities level. Network in *D* shows the time effect on network dynamics in a long duration recording. Without any pharmacological treatment network maintained similar firing and bursting patterns as shown in the interval of 1 h. Network in *A*, after treatment with DL-TBOA (10 μM), resulted in prolonged and frequent SBs. The dormant states were shorter and contained with lesser asynchronous activities. Similar effects were observed after DHK (GLT-1 specific, 200 μM) treatment in network in *B*. The only difference between DHK and DL-TBOA was an increase of asynchronous activities during the dormant state under DHK. Specific augmentation of GLT-1 of network in *C* with GT949 (10 μM) resulted in shortening of SBs. Role of GLT-1 was further tested in disinhibited state of cortical networks. Disinhibition with Bic (GABA inhibitor, 10 μM), shown in network in *E*, resulted in more robust SB patterns with clear active and dormant states of network. Resulting SBs were longer than reference. Further DHK (200 μM) addition enhanced SB frequency and reduced SB duration at the same time. Enhancing GLT-1 function with GT949 (10 μM) treatment in disinhibited state (network in *F*), resulted in shorter SBs.

To gain further insights on the role of GluTs in SBs, we used pharmacological tools to dissect their contribution. Adding GluT-specific inhibitor drugs resulted in dramatic change in firing and bursting patterns (Fig. 4*A*,*B*). Inhibition with DL-TBOA resulted in 2-folds increase in array-wide firings (Table 1). Still there was no significant change in SB frequency. The SB duration was increased nearly 2.5 times. The level of array-wide spiking activities during SBs was quantified as SB index (ranging between 0 and 1, see Material and Methods section for more information). Our cultured networks usually displayed an SB index of ≈0.8 in their reference recordings. DL-TBOA treatment significantly increased SB index. We also observed a rearrangement effect of firing activities in different samples. Although all networks showed consistent increase of firing activities after DL-TBOA addition, a range of change in SB duration was observed across samples. Networks that showed small increases in burst duration after the drug treatment showed increased frequency of SB compared with their reference, whereas networks that displayed big increase in burst duration showed a decrease in SB frequency. This rearrangement effects were possibly due to variability in development of connection topology across culture preparations. Specific inhibition of GLT-1 with DHK reproduced the effects similar to DL-TBOA treatment. The only difference was found in the SB index of the networks after drug treatment. Contrary to DL-TBOA effect, DHK addition resulted in increased asynchronous state firings that significantly reduced the burst index (Table 1). On the other hand, augmenting GLT-1 function with GT949, a PAM drug, significantly decreased array-wide firings without affecting SB frequency and SB index. The duration of SBs became shorter than their reference, a consequence likely due to reduced firing activities (Fig. 4*C*). These results clearly show that the synaptic efficacy during SBs is regulated by GLT-1.

**Table 1:** Pharmacological targeting GluTs alters SB statistics

Drug treatments	Firing rate (spikes/s)	SB rate (min^−1^)	SB duration (s)	SB index (0–1)
Ref + DL-TBOA	25 ± 13 50 ± 30^**^ n = 13	6 ± 5 5 ± 4, n.s *n* = 16	0.28 ± 0.09 0.72 ± 0.44^***^ n = 16	0.75 ± 0.12 0.84 ± 0.11^*^ n = 13
Ref + DHK	26 ± 8 46 ± 13^***^ n = 11	6 ± 5 6 ± 6, n.s *n* = 11	0.34 ± 0.17 0.66 ± 0.39^**^ n = 11	0.84 ± 0.12 0.67 ± 0.18^**^ n = 10
Ref + GT949	29 ± 14 15 ± 10^***^ n = 14	5 ± 2 4 ± 2, n.s *n* = 11	0.27 ± 0.12 0.18 ± 0.06* n = 9	0.80 ± 0.16 0.83 ± 0.09, n.s *n* = 12

Notes: Summary of the changes in array-wide neuronal activities from reference (Ref) after pharmacological treatments with GluT inhibitors; DL-TBOA(10 μM), DHK(200 μM), or selective augmentation of GLT-1 GluT with PAM drug GT949 (10 μM). n.s = not significant.

Statistical tests: paired *t* tests, significance with Ref as baseline was defined as ^*^P < 0.05, ^**^P < 0.01, and ^***^P < 0.001. *n* denotes number of cultures.

### SBs Are Not Terminated by Inhibition

Inputs from the inhibitory subnetworks are known to regulate the excitability of neurons. It is the interaction between the 2 that is believed to determine the flow of information through the network as well as shape network activities (Kudela et al. 2003; Ziburkus et al. 2006; Chen et al. 2006; Iida et al. 2018). To gain insight on the contribution of inhibitory inputs in SB, cortical networks were treated with Bic (GABA antagonist, 10 μM) to suppress the fast inhibitory effects of GABA. After Bic addition, the SBs became more periodic and longer (Fig. 4*E*,*F*). Networks in disinhibited state also often displayed reverberatory activities (sub-bursts) within each SB event similar to previous report of (Lau and Bi 2005). Asynchronous activities were almost abolished while SBs were more prominent, with rather clearly maintained active and dormant phases. The observation that networks did not fire endlessly and stop until there is no more activity after Bic treatment shows that the mechanism of SB termination is not limited to excitatory–inhibitory interactions.

Analyzing the firing patterns, cultures treated with Bic showed significant increase in firing rate (Table 2). After disinhibition, networks engaged only in synchronous activities (burst index ≈ 1). Disinhibition also resulted in significant increase in the duration of SB while maintaining similar SB frequency compared to reference. To validate the role of GLT-1 in regulation of SB dynamics even in disinhibited state of cortical networks, we further pharmacologically manipulated GLT-1 transporters in the disinhibited state of network. Further addition of DHK did not affect the overall firing rate or the SB index but induced a reorganization of the firing events into shorter duration with higher frequency of bursting (Fig. 4*E*). These effects were reversible after washout of the drugs (data not shown). Adding GT949 in the disinhibited networks significantly decreased the overall network firing activities, while maintaining the same high SB index induced from disinhibition (Fig. 4*F*). Under the influence of GT949, burst duration was significantly shorter, without affecting the SB frequency (Table 2).

**Table 2:** Comparison of SB statistics under GLT-1 specific treatments in disinhibited state of cortical networks

Drug treatments	Firing rate (spikes/s)	SB rate (min^−1^)	SB duration (s)	SB index (0–1)
Ref + Bic + DHK	34 ± 22 82 ± 48^**^ 97 ± 31, n.s *F*(2,10) = 21.19^***^	7 ± 5 7 ± 4, n.s 21 ± 16^*^ F(2,10) = 10.26^**^	0.28 ± 0.19 0.85 ± 0.49^**^ 0.58 ± 0.35, n.s *F*(2,8) = 12.21^**^	0.86 ± 0.12 0.99 ± 0.01* 0.99 ± 0.01, n.s χ^2^(2,9) = 9.8^**^
Ref + Bic + GT949	27 ± 17 60 ± 42^*^ 12 ± 08^**^ F(2,9) = 14.88^**^	5 ± 2 8 ± 4, n.s 5 ± 3, n.s *F*(2,8) = 2.43, n.s	0.35 ± 0.24 0.73 ± 0.42^***^ 0.17 ± 0.05^**^ F(2,9) = 16.64^**^	0.84 ± 0.12 0.99 ± 0.01** 0.99 ± 0.02, n.s χ^2^(2,8) = 12.67^***^

Notes: Summary of the changes in array-wide neuronal activities from Ref to pharmacologically disinhibited state (Bic, 10 μM) followed by manipulation of GLT-1 GluTs with DHK (200 μM) or GT949 (10 μM). n.s = not significant.

Statistical tests: repeated measures 1-way ANOVA (*F*) and nonparametric Friedman test (χ^2^), significance ^*^P < 0.05.

### Synchronous Calcium Elevation in Astrocytes is Induced by SBs

**Figure 5:**
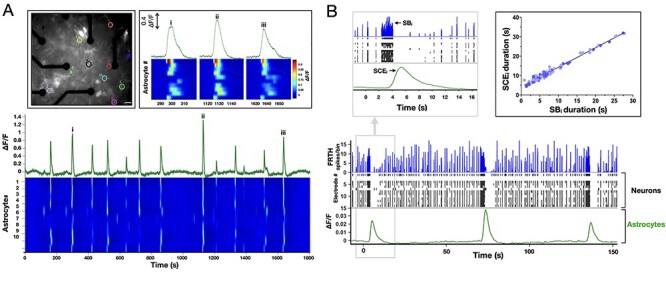
Astrocytes exhibit synchronous calcium dynamics following SBs. Top inset on left shows fluorescence micrograph of a culture expressed with cyto-GCaMP6f calcium sensors in astrocytes, scale bar = 30 μm. Different colored circles indicate locations of 10 randomly selected and spatially distant calcium-active astrocytes. Traces in green show summed fluorescence intensity–time plot of the selected astrocytes. Heatmap shows corresponding temporal dynamics of calcium intensity in individual astrocytes. Top right inset shows enlarged view of 3 SCE events marked as (i, ii, iii). Note that the activation pattern remains similar in each event, indicating a hierarchical order of astrocyte activation during SCEs. (*B*) Shows temporally aligned activities of neurons obtained from MEA (FRTH with 40-ms bin size, blue trace) and corresponding raster plot (black trace), with collective calcium activities in astrocytes. Top left inset shows enlarged view of a global event when an astrocytic SCE appears when a long persisting neuronal SB occurs. Note that all neuronal activities ceased during the global calcium rise in astrocytes. Neuronal activity reappeared soon after the SCE decay. Top right inset shows duration of SCE was found to be linearly related to its preceding SB duration (*n* = 87, 4 cultures).

It is known that astrocytes are calcium sensitive toward synaptic glutamate activities (Dani et al. 1992). Previous reports show that spontaneous activities in neurons and astrocytes are patterned into correlated neuronal/astrocytic networks in which neuronal activity regulates the network properties of astrocytes (Aguado et al. 2002). The role of astrocytic calcium activities in synaptic events still remains widely debated. Therefore, we further investigated astrocytic collective calcium dynamics in the cortical networks with respect to the spontaneous SBs in neurons. Cultures were expressed with genetically encoded calcium sensors (cyto-GCaMP6f) specific to the astrocytes. GCaMP6f imaging revealed asynchronous local calcium elevation as well as synchronous calcium elevations (SCEs, Fig. 5*A*). We noticed that the sequence of astrocytic activation during the global calcium elevation was not random. A hierarchy in the recruitment of calcium was consistently observed. This was likely due to an existence of a stable subset of privileged neurons whose spiking activities reliably increase before the onset of global SB (Eytan and Marom 2006). Combining MEA and calcium imaging, temporal activities from the 2 cell types were recorded simultaneously. Consistently, we observed that SCEs always emerged toward the end of longer persisting SBs (Fig. 5*B*). Note that during the SCE in astrocytes, neuronal ongoing activities were completely abolished. Neurons resumed their basal activity level shortly after decay of SCE in astrocytes. Importantly, only SBs persisting long enough (*>*500 ms) were followed by a global astrocytic SCE. Since SBs result in large glutamate transient at synapses globally, this transient is likely to trigger collective activation of astrocytic metabotropic glutamate subtype 5 receptors (Panatier et al. 2011). A positive correlation was found between the duration of SB in neurons and its concomitant SCE in astrocytes. To validate whether glutamate released by the SBs were responsible for SCE in astrocytes, cultures were treated with riluzole (inhibitor for glutamate release, 10 μM). Treatment with riluzole resulted in suppressed neuronal activities with complete abolition of SBs and associated SCEs (data not shown). Although neurons maintained some asynchronous firings, no astrocytic SCE was observed after addition of riluzole, indicating astrocytic dependency on neuronal SBs for generation of SCEs.

### Simulation Results

**Figure 6:**
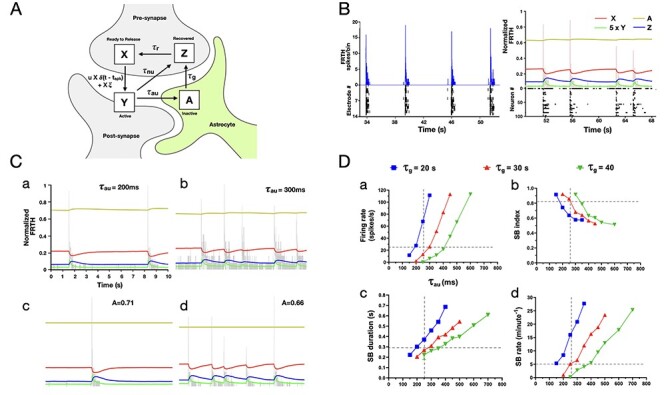
Astrocytes regulate glutamate availability in neurons. Schematic representation of TUMA, tripartite synapse model describing glutamate recycling is shown in (*A*). The fraction of glutamate in the different states are represented by *X* (in presynapse), *Y* (at synapse), *Z* (recovered at presynapse), and *A* (in astrocytes) as shown in the figure, where *X* + *Y* + *Z* + *A* = 1. Impulses δ(*t* − *t*_spk_) created by spikes at time *t* = *t*_spk_, arriving at the presynapse, trigger glutamate release into the cleft. Released glutamate molecules rapidly activate the postsynaptic fast receptors until their clearance by neuronal and astrocytic uptake with timescales τ_nu_ and τ_au_, respectively. The astrocytic uptake follows glutamate–glutamine cycle leading to slow recovery of glutamate at the timescale of τ*_g_* in the presynapse. Once recovered, glutamate is retrieved back at the active zone within τ*_r_*. During this recovery process, some neurons may exhibit asynchronous release induced by noise (ξ). (*B*, left) Shows FRTH (blue trace, bin size 5 ms) and raster plot (black trace) of neuronal activities obtained from an MEA recording. (*B*, right) Similar firing and bursting patterns were obtained from simulation (random network of 100 neurons connected with TUMA synapses). Simulated normalized FRTH trace (bin size 5 ms) is overlapped with the 4-state *X* (red), *Y* (green), *Z* (blue), and *A* (yellow) dynamics of glutamate occurring simultaneously in the network. These traces represent the average fraction of glutamate distributed around the synapses. Note that during SBs, a very small fraction of the total glutamate gets released. Therefore, *Y* is shown 5 times its actual value for visual clarity. (C) Shows the effects of varying the astrocytic uptake timescale τ_au_ on network firing patterns (FRTH, gray trace). Subplots (*a*–*d*) in (*C*) share the same vertical and horizontal axis scales with (*a*). Increasing τ_au_ from 200 to 300 ms resulted in longer and frequent SBs similar to DHK effects (*a*–*b*). Fxing *A* to the mean values (0.71 and 0.66) obtained from τ_au_ (200 and 300 ms) did not affect the SB patterns (*c*–*d*). The summary of network firing statistics dependence on astrocytic uptake τ_au_ and transport τ*_g_* timescales is shown in (*D*). Each data point represents the mean value obtained at the specified parameter values. Effects of varying τ*_g_*, that is, recovery time of glutamate from astrocytes, on SB properties is shown in color-coded traces. Overlapped vertical and horizontal broken gray lines represent the approximate mean value of glutamate decay time (τ_decay_) analog for astrocytic glutamate uptake (τ_au_) obtained from experiment (Fig. 3*C*) and the approximate SB statistical mean values obtained from MEA data analysis, respectively. The trends in simulation results were consistent with the trends observed in DHK experiments slower astrocytic glutamate uptake, that is, with increase in τ_au_ (*D*-*a*) the firing rate also increases, (*D-b*) SB index decreases, (*D-c*) SB duration increases, and (*D-d*) SB rate increases in the network. Moreover, the SB statistics were found to match with experimental observations closely at τ*_g_* around 30 s.

The description of firing and bursting patterns obtained from MEA experiments show how the dynamics of SB changes under various conditions. We used these firing patterns as input to obtain relevant parameters for our tripartite synapse TUMA model. This model describes how glutamate gets relocated to different parts of a tripartite synapse. For model details, see Material and Methods section. The essentials of this model can be understood from the schematic diagram shown in Figure 6*A*. The amount of glutamate (expressed as fractions) in the system can be in *X* (ready-to-release pool), *Y* (active state; producing EPSC), *Z* (pool of glutamate uptaken from the synapse), and *A* (glutamate uptaken by astrocyte). Similar to the TUM model (Tsodyks et al. 2000), we implemented conservation of glutamate in the tripartite synapse; with *X* + *Y* + *Z* + *A* = 1. The GluTs targeted in this study are related to the 2 uptake timescales in TUMA synapse; τ_nu_ (neuronal uptake) and τ_au_ (astrocytic uptake). The values of τ_nu_ and τ_au_ were obtained and fixed in a manner to match the simulated firing patterns with the experimental observations. These time constants and the model mechanism were the basis of our understanding on how glutamate trafficking dynamics regulate the SB properties.

#### SB Events

Figure 6*B* shows the raster plot of typical noise-induced synchronous firing events in 100 neurons randomly connected through TUMA synapses. The time course of *X*, *Y*, *Z*, and *A* state of glutamate averaged across all synapses during the SB events are also shown in the figure. Simulation parameters (Table 3) were chosen such that the firing patterns were similar to experimental firing patterns and consistent with previous reports (Volman et al. 2007; Huang et al. 2017b). Prior to the onset of SB, fraction *X* remains at its maximum whereas *Z* at its minimum. Most of the changes in *X* get supplied from *Z*. Once the SB is triggered, a steep rise in *Y* happens similar to the experimental observations (Fig. 3*B*). One remarkable feature shown in the figure is that during an SB, changes in *X* and *Z* are large while only very small changes occurs in *A*. These changes in *A* during SB are small because of the slow process for *A* to turn into *Z*. If this later process could be faster, one would expect bigger changes in *A* during an SB event.

**Table 3:** List of parameters used in simulation

*g*Ca	1.1 mS.cm^−2^	*C*	1 μF cm^−2^	*k_a_*	0.1 μM
*g_K_*	2 mS.cm^−2^	*υ*	0.2 m s^−1^	*k_r_*	0.4 μM
*g_L_*	0.46 mS.cm^−2^	*I*_bgm_	27 μA	β	5 μM s^−1^
*V*_Ca_	100 mV	*I*_bgs_	0 μA	*m*	4
*V_K_*	−70 mV	*u*_low_	0.2	*n*	2
*V_L_*	−65 mV	*u*_up_	0.2	*I_p_*	0.11 μM s^−1^
*V*_1_	−1 mV	τ_nu_	50 ms	γ	50 μM s^−1^
*V*_2_	15 mV	*τ_r_*	600 ms	[Ca2+]*_o_*	2 mM
*V*_3_	0 mV	τ_au_	250 ms	*V*_th_	10 mV
*V*_4_	30 mV	τ*_g_*	30 s	*w*_ee_	4.0
*V_r_*	0 mV	ξ_ave_	0.02	*w*_ei_	4.0
*V_i_*	−90 mV	η_max_	0.32 ms^−1^	*b*	0

Notes: With the exceptions of τ_au_ and τ*_g_*, all the other parameter values used in this work were obtained from previous works (Tsodyks et al. 2000; Volman et al. 2007; Huang et al. 2017b). Throughout all the simulation runs, the leaking channel conductance parameter (*g_L_*) was set slightly lower (0.46 mS) than the previous works (0.5 mS) to induce more spiking activities. The value of release constant for residual calcium (γ) was used as 33 μM s^−1^ (Huang et al. 2017b), whereas (Volman et al. 2007) used 80 μM s^−1^. In our simulations γ was tuned to 50 μM s^−1^. After fixing the astrocytic uptake time constant τ_au_ equal to the experimentally obtained τ_decay_ value (see Fig. 3*C*), glutamate recovery to the *Z* state by the neuronal uptake mechanism τ_nu_ was tuned to 50 ms to match with the experimental firing and bursting patterns. Glutamate retrieval from the *Z* state to the ready to release active zone *X* was set (600 ms) within the range of “kiss-n-run” mode of vesicle retrieval τ*_r_* (Gandhi and Stevens 2003). All the other remaining parameters were identical to the previous works (Tsodyks et al. 2000; Volman et al. 2007; Huang et al. 2017b).

#### Slower Astrocytic Uptake Alters SB Features

The effects of the astrocyte uptake timescale on the properties of SB are shown in Figure 6*C*(*a*,*b*). An increase in τ_au_, from 200 to 300 ms, resulted in a substantial increase in the overall firings, bursting rate, bursting duration, and burst index (Fig. 6*D*). These effects are similar to the inhibition of the astrocytic uptake by DHK treatment in our experiments. The increases in the SB statistics reflect that more glutamate becomes available in the synapse while *A* gets smaller. Figure 6*C*(*a*,*b*) shows that *A* remains relatively constant during the SBs; however, its mean value is sensitive to the value of τ_au_. A change of τ_au_ from 200 to 300 ms decreases *A* from 0.71 to 0.66. Further increase in τ_au_ would decrease *A* to an even smaller value. These findings are consistent with the schematic picture that *A* works as a temporary storage for the glutamate and can regulate the amount of available glutamate in the synapse (Jabaudon et al. 1999). With the parameters used to reproduce experiment observations, the simulation revealed that *A* remains relatively constant and sets the amount of glutamate available to *X*.

#### SB Can Be Produced With Fixed Amount of Glutamate in Astrocytes

The value of *A* remains nearly constant during SBs for different values of τ_au_ as shown in Figure 6*C*(*a*,*b*). These results indicate that astrocytes do not directly take part in the generation of an SB. Their primary role is to restrict the amount of glutamate available for synaptic dynamics. By fixing *A* as a constant (Fig. 6*C*[*c*,d]), SBs can still be generated with features similar to the experiments, indicating that the dynamics of *A* may not be important for the generation of SB. By fixing *A*, the tripartite synapse in fact gets reduced into a bipartite synapse. As a bipartite (TUM) synapse is governed by *X* + *Y* + *Z* = 1, fixing *A* was equivalent to *X* + *Y* + *Z* = 1 − *A*, where 1 − *A* became another constant, which is *<* 1. This strongly suggests that the main role of astrocytes in generation of SB is to limit the amount of glutamate available in the bipartite synapse. The availability of glutamate then governs the firing patterns in SBs.

#### Slower Glutamate Recovery From Astrocytes in Neurons Alters SB Properties

According to our TUMA model, the amount of glutamate in the *A* state can be varied either by varying τ_au_ or τ*_g_*. Here, τ*_g_* represents the timescale for glutamate–glutamine cycle, which is followed by astrocytic glutamate uptake. Previous lines of research have shown the importance of this cycle for the generation of bursting activities in neurons (Bacci et al. 2002; Tani et al. 2014). Figure 6*D* shows network bursting statistics under combinations of different τ_au_ and τ*_g_*. Although here τ*_g_* has been used as an overly simplified representation of multiple processes that occur in the course of glutamine transport from astrocytes to neurons, these simulation results provide an estimated range of τ*_g_* within which this recovery may take place. From the data pooled from our cortical culture experiments, the reference SB statistics were found to be in the following ranges: array-wide firing rate (≈26 spikes/s), SB duration (≈0.29 s), SB index (≈0.82), and SB rate (≈5 SBs/min). Also, the glutamate decay time (τ_decay_) measured across networks was found to be ≈0.26 s. In order to align with these values, it can be seen in Figure 6D that τ*_g_* should lie around 30-s range. Also, the effect of increasing τ*_g_* on bursting activities were in line with the effect of blocking astrocytic glutamate–glutamine cycle, as shown in the previous reports (Bacci et al. 2002; Tani et al. 2014).

## Discussion

SB is a network phenomenon and believed to be related to the proper or malfunction of the network. Although SB has been observed for a long time, its fundamental mechanism is still not clear. With our experiments and modeling, it is clear that the SB is the result of the interaction between the neuronal and glial systems in the network. From the results shown above, the properties of SBs can be altered by local changes of glutamate dynamics in the level of individual synapses. The local changes are referred to the glutamate recycling at a synapse by astrocytes. Previously, glutamate recycle via glutamate–glutamine cycle has been shown to be necessary for sustained neurotransmissions (Bechtholt-Gompf et al. 2010; Tani et al. 2014). Here we experimentally tested the role of glutamate uptake mechanism in SB, which essentially precedes the glutamate–glutamine cycle within the recycle mechanism. We extended our findings with the mentioned previous works in simulation to investigate the overall effect of local recycle on a global phenomenon, that is, SB. The local recycling process is synchronized in the network through the neural activity. During SB, only the envelope of bursts is synchronized but not the individual spikes of the neurons (Huang et al. 2015). Therefore, there is global synchronization in the IGluSnFr fluorescence images that are sensitive only to the accumulation of glutamate during bursts (in order of 100-ms timescale). Local glutamate releases at different synapses are related to individual spikes (1-ms timescale) of different neurons whose detection require high sensitivity and speed of detection equipment. Unfortunately, florescence imaging with such high specifications was beyond the scope of our equipment.

Of particular importance is the GLT-1 GluTs expressed on the astrocytes that transport the majority of glutamate in the synapses into the astrocytes. We experimentally observed that cell nonspecific glutamate transport inhibition (with DL-TBOA) had effects similar to specific inhibition of astrocytic GLT-1 (with DHK), strongly indicating that astrocytic glutamate clearance control SB dynamics. The only difference between the 2 treatments was found in the SB index, arising from the difference in neuronal activities during the dormant phase. DHK treatment resulted in more asynchronous firings giving a lower SB index, whereas DL-TBOA resulted in less asynchronous firings during the dormant phase giving a higher SB index. With respect to our TUMA model, the effect of DHK (astrocytic GLT-1 uptake inhibitor) is an increase in τ_au_, whereas the effects of TBOA (astrocytic and neuronal GluT inhibitor; Shimamoto et al, 1998) are increases in both τ_au_ and τ_nu_. Increasing the τ_au_ parameter in simulation successfully reproduced all trends of DHK treatment correctly. But increasing the τ_nu_ and τ_nu_ parameters simultaneously could not reproduce all the effects of TBOA treatment. Increasing the 2 parameters at the same time gave the correct trend of increase in firing rate and SB duration but failed to reproduce the correct SB index trend. Increments in the 2 parameters further enhanced the asynchronous firings during the dormant phase. This difference in simulation and experimental observation suggests that TBOA treatment might also be affecting other presynaptic mechanisms that regulate spontaneous releases. Presumably, there are still other trafficking mechanisms at the presynaptic terminal that are not accounted for in our model. This shortcoming of our model needs to be improved in the future. Despite this limitation, TUMA model clarifies that the local changes in the transportation of glutamate in the synapses are manifested as the changes in the properties of the SB in the network. Therefore, the global changes of SB in the network level observed in our experiments can be used to infer useful information on the local glutamate transport; especially with the participation of the astrocytes in a tripartite synapse.

In our TUMA model, the SBs originate from the asynchronous releases of glutamate from neurons and the effects of these random events get amplified by the positive feedback of the recurrent network in the culture as elevated firing spreading across the network. Our important finding is that the astrocyte and its glutamate handling properties are playing a crucial role of arresting this rapid network firing by providing a negative feedback in reducing the availability of glutamate in the neuronal system. The observed SB is then the result of the sequential manifestation of the positive and negative feedback in the neuronal and glial systems, respectively, in the network. Details of the SB mechanism in our TUMA model are described below.

### Initiation and Positive Feedback

In the dormant phase of the network, the number of asynchronous firings increases (Fig. 2*A*) before SB. These firings originate from the noise-induced release of *X* that build up slowly during the dormant state. If these release events become large enough and the connectivity in the network is also high enough, action potentials gets triggered in some neurons. Once this happens, more glutamate gets released by these action potentials triggering more neurons to fire due to recurrent connections in the system. Consequently, this positive feedback then triggers a system-wide firing event.

### Maintenance of Burst (Active Phase)

The positive feedback can be maintained as long as there is enough *X* to be released to trigger further action potentials. Depending on the system parameters (*A* level), sub-burst in the form of reverberations can also be possible because of the interaction between synaptic facilitation and depression in the TUM mechanism. One would expect reverberations to be more easily observed in a system with only excitatory inputs similar to experiments with Bic. This is because for systems with both inhibitory and excitatory inputs, there should be a wider and weaker distribution of input currents to the neurons. Intuitively, system with higher *X* (lower *A*) should have longer bursting duration because it will take longer for *X* to deplete to a certain threshold. Indeed, we see from both experiments (Table 1) and simulations (Fig. 6*C*) that SB duration decreases with the amount of glutamate in the astrocyte.

### Negative Feedback and Termination of Burst

During the active state of SB, the ready-to-release glutamate molecules (*X*) are constantly being transformed into *Z*, via multiple timescale processes, which will then be turned slowly back to *X* controlled by another long time constant. Due to overall slower recovery mechanism, *X* is not replenished fast enough from *Z*. The action potential triggered release from the presynaptic cell will be then too small to elicit action potentials at the postsynaptic cell. In this scenario, the positive feedback can no longer be sustained and the bursting will stop.

### Dormant Phase (Inactive Phase)

Once the SB terminates, the system enters into a dormant phase in which the system-wide firings stop and there are only isolated spikes created by noise-driven releases. During this phase, *X* can slowly recover from *Z,* which is now dependent on very slow recovery from *A*. As *X* increases, the amount of noise-driven-released glutamate will also increase, raising the probability of triggering a system-wide depolarization.

The TUMA model can be further extended to include more details on glutamate and glutamine pools transitions in both neurons and astrocytes. To include such details, more experimental evidence and related timescales need to be established. These experiments are beyond the scope of our current study. The transition details can be introduced by creating additional variables between the *A* state in astrocyte and *Z* state in neuron. Such extensions of the current model would have basically introduced more timescales (pool transition associated) between the 2 states. As experimental evidence on these timescales remain obscure, currently the pool transition processes are lumped into single very slow timescale (τ*_g_* = 30 s). Notably, the excitability of neurons primarily depends on *X*. Hence, inclusion of the new variables would although provide more information on pool trafficking dynamics, the overall effect on SB generation should still remain the same.

With the picture described above, the SB phenomenon can be considered as a generic emerging property arising from the negative and positive feedback interaction between the glial and neuronal networks. No special circuits (or buster neurons) or disinhibition (Fig. 4*E*,*F*), the 2 traditional bursting mechanisms, are needed for its generation. The most important finding in our work is that the firing and bursting patterns of the neurons are governed by the amount of glutamate in the astrocytes (*A*). It is possible that different regions of brain can have different amount *A* in the astrocytes locally, allowing different firing and bursting patterns at different brain regions. In this sense, the astrocytes are regulating the bursting property of neurons in the network.

As shown in the simulation results, the astrocytes maintain a much higher level of glutamate than neurons. This trafficking arises due to the simultaneously occurring fast synaptic glutamate uptake via its transporters and slow glutamate–glutamine cycle processes. This mismatch between the 2 timescales in effect results in accumulation of higher amount of glutamate in the astrocytes. Hence, the level of glutamate availability in the presynaptic neuron gets restricted. Presumably, for normal function of the neural network, the amount stored in or released from the astrocytes are adaptive to the problem at hand. A low astrocytic glutamate concentration is necessary for reliable synaptic transmission of information (Flanagan et al. 2018) and our case of high glutamate concentration in astrocytes during SB is consistent with the enhanced glutamate levels found in epilepsy. Arguably, the SBs observed in our experiments are similar to pathological states of the system.

Similar to the TUM model, it was assumed in our TUMA model that the amount of glutamate remains conserved within the tripartite synapse. This assumption may not be true since the glutamate–glutamine cycle is an open-ended cycle and astrocytes can metabolize glutamate from another pathway involving tricarboxylic acid cycle (Eid et al. 2016). Moreover, astrocytes are reported to perform intercellular glucose trafficking (Rouach et al. 2008). So heterosynaptic redistribution of glutamate via astrocytes could be possible. But during the different phases of SB, a very small change occurs in *A* (Fig. 6). In fact, if the amount of glutamate in the *A* state is fixed, qualitatively similar collective bursting patterns can still be obtained (Fig. 6*C*). If *A* is fixed, we are back to the traditional bipartite synapse (a TUM model with *X* + *Y* + *Z* being a constant but less than one). Functionally, the astrocytes set the overall level of *A* through the 2 processes of uptake from *Y* and transforming *A* to *X*. Therefore, even if there is a violation of the conservation, it will not invalidate our conclusion as long as the overall *A* is fixed to a certain level.

During the active state, it is clear that the astrocytes exhibit SCEs (Fig. 5). Its effect was not considered in our TUMA model. From Figure 5*B*, the SCEs in astrocytes were induced toward the termination of SBs. Longer SBs more reliably triggered SCEs in astrocytes than shorter SBs. This observation indicates that astrocytic global calcium response depends on the amount of glutamate released in synapses. Interestingly, during the SCE transient in astrocytes, all the neurons maintained complete quiescence. Some electrodes that were tonically active during both the active and dormant states were also found to be quiescent during the SCE in astrocytes. The neuronal firing activities recovered shortly after the astrocytic calcium started decaying. For such an observation, there could be multiple possible scenarios: First, persisting SBs activate calcium elevation in astrocytes that may result in release of astrocytic gliotransmitters. Released gliotransmitter may then directly act on the neuronal receptors to suppress the persisting neuronal activities (Montana et al. 2006). Two possible candidates are ATP/adenosine (Zhang et al. 2003; Panatier et al. 2011) and nitric oxide (Mehta et al. 2008) that have been reported to suppress heterosynaptic activities. Second, the network may enter into a “refractory” phase due higher depletion of presynaptic glutamate during longer persisting SBs. However, under physiological conditions the presynaptic pools in neurons do not completely deplete (Harris and Sultan 1995; Schikorski and Stevens 1997). Therefore, we think that the former hypothesis could be the case in cultured cortical networks. Up till now, we have been discussing the effects of negative feedback from astrocyte. It is also known that astrocytic calcium elevation can enhance the excitability (through facilitation) of the postsynaptic cells in the network (Verdugo et al. 2019). Concomitant astrocytic SCE response could be associated with positive feedback mechanism to the neurons through calcium-induced glutamate release (Parpura and Haydon 2000). It requires further more experiments to carefully dissect and confirm these speculations.

Finally, we like to point out that malfunction of astrocytic GLT-1 is related to neurological disorders such as seizure-like epilepsy (Tanaka et al. 1997; Werner et al. 2001) as well as behavioral disorders (Bechtholt-Gompf et al. 2010; John et al. 2012; Scofield and Kalivas 2014). Moreover, electrophysiological evidence shows that astrocyte-dependent glutamate recycling is important for active neurotransmissions (Tani et al. 2014). Our study in consolidation with previous reports clarifies the understanding of how altered uptake mechanisms or astrocyte-dependent glutamate recycling can affect neuronal network firings and collective behavior, which are representative of brain function as well as dysfunction. Previous astrocyte-targeted in vivo studies involving DHK treatment has been shown to induce depression-like (anhedonia) behavior in rats (Bechtholt-Gompf et al. 2010; John et al. 2012). With the same drug used in this study, TUMA model helps in explaining some aspects of their findings, such as increase in neuronal activities after DHK treatment. If we extend our findings to understand their observations, it seems that the increase in firings induced by the DHK is the origin of such changes. In terms of our model, this is just a decrease in the level of glutamate in the astrocytes (the *A* state). This last observation is consistent with the current view that astrocytes can be important in shaping the behavior of animals (Harada et al. 2016).

## Notes

*Conflict of Interest*: None declared.

## Funding

Taiwan’s Ministry of Science and Technology (MOST) (105-2112-M001-017-MY3) and (108-2112-M-001-029-MY3).
